# Coaching Servant Leadership: Scale Development and Validation

**DOI:** 10.3389/fspor.2022.871495

**Published:** 2022-05-18

**Authors:** Shohei Takamatsu

**Affiliations:** Faculty of Education, Kobe Shinwa Women's University, Kobe, Japan

**Keywords:** servant leader, leadership, coach, scale development, athlete first, sport, high school, university

## Abstract

This study aimed to develop a valid and reliable scale for measuring coaching servant leadership in different contexts (Japan and the United States). First, potential items were collected in Japan using both deductive (i.e., literature review) and inductive (i.e., surveys among 103 coaches and 34 university students) approaches and narrowed down via content validity assessment by 10 experts. Next, quantitative studies were conducted to validate the scale's construct validity, among 936 high school athletes from Japan. Finally, the scale's applicability to the US context was demonstrated, among 278 university athletes in the US. The analyses resulted in a six-factor model with 17 items to assess coaching servant leadership behaviors: (1) acceptance; (2) shared vision; (3) empowerment; (4) dedication; (5) humility; and (6) winning second. In conclusion, this study developed a coaching servant leadership scale by applying both deductive and inductive approaches and deemed it applicable not only in Japan but also in the US. It is anticipated that future studies will examine the impact of coaching servant leadership on athletes in detail, with findings applied in practice for the development of coaches.

## Introduction

Fifty years have passed since Greenleaf (1970) introduced the servant leadership concept. Servant leadership is a leadership approach that promotes authenticity, focuses on supporting followers, and prioritizes maximization of followers' potential (Liden et al., 2015). According to Rieke et al. (2008), the leader is traditionally at the top of the “pyramid,” with followers are expected to follow their directions; however, a servant leader inverts the pyramid and places themselves at the bottom of the hierarchy. In a servant leadership environment, followers are given clear roles, and the leader's job is to help them perform these roles (Rieke et al., 2008). Several researchers have developed scales to measure servant leadership: Eva et al. (2019), for example, reviewed 16 such measurement scales and clarified the antecedents, moderators, mediators, and outcomes of servant leadership by reviewing empirical studies on servant leadership. Many studies have focused on the business management field.

The sport domain has witnessed increasing interest in servant leadership. Sullivan (2019) highlighted several reasons for this, including the fact that many schools belonging to the National Collegiate Athletic Association (NCAA) have been punished for violations. Moreover, values have changed with the growth in the number of working millennials (i.e., those born between 1983 and 1994), and positive psychology, including the concept of wellbeing, has increased in popularity. Since the NCAA's core values include integrity and sportspersonship, respect, and inclusive cultures (Robinson et al., 2018), servant leadership—an other-centered approach that prioritizes followers' needs, growth, and well-being—is considered to play a vital role. Hammermeister et al. (2008) examined the applicability of the revised servant leadership profile (Wong, 2004) in the sport domain. Although many similarities between business and sport have been acknowledged (Rieke et al., 2008), the unique servant leadership of coaches remains insufficiently examined (e.g., the servant leadership of coaches may be similar to the coaching philosophy of “athlete first, winning second”). Consequently, sport researchers have used instruments from different fields, and few studies have examined the specific effects of coaches' servant leadership (Hammermeister et al., 2008). Hammermeister et al. (2008), who examined a servant leadership scale consisting of three factors, highlighted the need for a new scale to explore other potential factors in coaching servant leadership. In developing a scale to measure coaches' servant leadership, the causal relationships between various concepts may be more specifically examined and compared according to culture and personal demographics. By clarifying the factors that constitute coaches' servant leadership, coaching that is implemented with the athlete's perspective in mind can be extended in practice. Therefore, this study aimed to develop a valid and reliable scale for measuring coaching servant leadership. First, leadership concepts that are similar to servant leadership (i.e., transformational leadership and authentic leadership) and servant leadership were reviewed, and four phases were identified to develop a scale.

## Literature Review

### Transformational Leadership

The transformational leadership concept is most frequently compared to servant leadership (Sullivan, 2019). According to Parris and Peachey (2012), Downton (1973) first mentioned transformational leadership, and Burns (1978) drew a distinction between transformational and transactional leadership in a political context. Bass (1985) then conceptualized this by applying transformational leadership to the organizational context. Transformational leaders encourage their followers to achieve substantial results and enhance their leadership capacity in the process (Bass and Riggio, 2006). Transformational leaders help followers develop by responding to their needs, empowering them, and aligning them with their goals and the goals of the organizations (Bass and Riggio, 2006). Bass's model (i.e., the Multifactor Leadership Questionnaire; MLQ) of transformational leadership consists of four I's (Bass, 1985): idealized influence, inspirational motivation, intellectual stimulation, and individualized consideration. While Bass's model has primarily been used to evaluate transformational leadership (Van Knippenberg and Sitkin, 2013), it has been revised to MLQ-5X (Bass and Avolio, 1995), which comprises five factors: Idealized influence (attributed); idealized influence (behaviors); inspirational motivation; intellectual stimulation; and individualized consideration.

The MLQ has also been used in the sport domain since the 1990s (Doherty and Danylchuk, 1996) and has been applied in many studies (Charbonneau et al., 2001; Rowold, 2006; Lee et al., 2013; Price and Weiss, 2013; Kao et al., 2019). In addition to the MLQ, the Differentiated Transformational Leadership Inventory (DTLI; Hardy et al., 2010) has been widely used in the sport domain. This is because Callow et al. (2009) modified the DTLI for the sport context and found that it is composed of seven factors: individual consideration, inspirational motivation, intellectual stimulation, fostering acceptance of group goals and teamwork, high performance expectations, appropriate role model, and contingent reward. Callow's et al. (2009) DTLI is related to many constructs, mainly in the sport psychology field (Arthur et al., 2011; Smith et al., 2013; Vella et al., 2013; Cronin et al., 2015).

Although the idealized influence and intellectual stimulation aspects of transformational leadership are similar to those of servant leadership (Liden et al., 2008), the differences between transformational leadership and servant leadership has been highlighted by several researchers. For example, Parolini et al. (2009) focused on five points—the moral, focus, motive and mission, development, and influence distinctions—and identified the differences between transformational leadership and servant leadership. Specifically, they emphasized the difference between the moral disadvantage of transformational leaders' focus on organizational goals and the moral advantage of servant leaders' focus on their individual followers' needs. Graham (1991) also highlighted the positive and negative aspects of individualized consideration and intellectual stimulation that ensue when leaders neglect their followers' moral development. In brief, transformational leaders are motivated by organizational development, while servant leaders are motivated by their followers' growth. Regarding these differences, Van Dierendonck et al. (2014) empirically verified that servant leadership is significantly associated with the satisfaction of followers' psychological needs while transformational leadership is not.

### Authentic Leadership

The conceptualization of authentic leadership by Luthans and Avolio (2003) and the detailed description of the components by Avolio and Gardner (2005) have been instrumental in the development of authentic leadership. Further refinements by Walumbwa et al. (2008) led to the most commonly used definition of authentic leadership (Banks et al., 2016) as “a pattern of leader behavior that draws upon and promotes both positive psychological capacities and a positive ethical climate, to foster greater self-awareness, an internalized moral perspective, balanced processing of information, and relational transparency on the part of leaders working with followers, fostering positive self-development (p. 94).” The authentic leadership questionnaire (ALQ) developed by Walumbwa et al. (2008) comprises four factors: self-awareness, relational transparency, internalized moral perspective, and balanced processing. Neider and Schriesheim (2011) supported the ALQ's construct validity and developed the authentic leadership inventory (ALI) based on Walumbwa's et al. (2008) theoretical framework and construct definitions. While few studies have examined authentic leadership in sport, ALQ has been used to verify the effects of head coaches' authentic leadership on athletes' psychological capital and team engagement (McDowell et al., 2018) and on athletes' autonomy, trust in their coach, enrollment, and commitment (Bandura and Kavussanu, 2018). Kim et al. (2017) applied ALI to examine the effect of head coaches' authentic leadership on assistant coaches' psychological capital, job satisfaction, and life satisfaction.

Authentic leadership and servant leadership share several similarities. Lemoine et al. (2019) identified moral behavior and the enhancement of followers' personal growth as commonalities between the two. While both leadership styles positively impact followers, servant leaders are more strongly motivated to serve others (Sullivan, 2019). Authentic leaders are interested in self-awareness and self-coordination, while servant leaders focus on the interests of others (Lemoine et al., 2019). Being true to oneself is not entirely consistent with the nature of servant leadership. Authentic leaders may feel a strong sense of responsibility for developing their organizations rather than focusing on their followers' needs and development (Robinson et al., 2018).

### Servant Leadership

The servant leadership approach consists of multiple dimensions (e.g., rational, relational, ethical, emotional, and spiritual) that empower followers to grow (Sendjaya, 2016; Eva et al., 2019). Greenleaf (2007) stated, “The servant-leader is servant first. It begins with the natural feeling that one wants to serve, to serve first. Then conscious choice brings one to aspire to lead” (p. 83). Servant leaders perceive the success not only of the organization but also of all their stakeholders as their moral responsibility (Greenleaf, 2002).

According to Eva et al. (2019), the existing research on servant leadership can be categorized into three phases. The first phase is the development of the concept by Greenleaf (1970) and Spears (1998). For example, Spears (1998) identified 10 characteristics of servant leadership: listening, empathy, healing, awareness, persuasion, conceptualization, foresight, stewardship, commitment to the growth of people, and building community. The second phase focuses on scale development and examines the relationship between servant leadership and other constructs. More scales have been developed for servant leadership scales than for other leadership styles (Laub, 1999; Page and Wong, 2000; Ehrhart, 2004; Barbuto and Wheeler, 2006; Liden et al., 2008; Sendjaya et al., 2008; Van Dierendonck and Nuijten, 2011). However, Eva et al. (2019) reviewed existing servant leadership scales in terms of Hinkin's (1995) scale development procedures (i.e., item generation, content adequacy assessment, questionnaire administration, factor analysis, internal consistency assessment, construct validity, and replication) and recommended the global servant leadership scale (SL-7; Liden et al., 2008), the servant leadership behavioral scale (SLBS-6; Sendjaya et al., 2008), and the servant leadership survey (SLS; Van Dierendonck and Nuijten, 2011), which implemented all of these procedures (see Table 1). Servant leadership research is currently entering its third phase, in which the focus has shifted toward the development of the theoretical model.

**Table 1:** Three servant leadership scales recommended by Eva et al. (2019).

**Authors**	**Name of scale**	**Number of factors and items**	**Factors**	**Sample**
Liden et al. (2008)	Global servant leadership scale (SL-7)	7 factors (28 items)	Emotional healing Creating value for the community Conceptual skills Empowering Helping subordinates grow and succeed Putting subordinates first Behaving ethically	Two samples • University students • Company employees and supervisors
Sendjaya et al. (2008)	Servant leadership behavioral scale (SLBS-6)	6 factors (35 items)	Voluntary subordination Authentic self Covenantal relationship Responsible morality Transcendental spirituality Transforming influence	One sample • Graduate students
Van Dierendonck and Nuijten (2011)	Servant leadership survey (SLS)	8 factors (30 items)	Empowerment Accountability Standing back Humility Authenticity Courage Forgiveness Stewardship	Eight samples • Participants of the open online survey • Participants of the open online survey • High school teachers • Combined sample from diverse occupations • Participants of the open online survey • Participants of the open online survey • Employees at gas stations • Online panelists

Hammermeister et al. (2008) surveyed collegiate athletes and developed the revised servant leadership profile for sport (RSLP-S), which consists of three factors: trust/inclusion, humility, and service. An overview of studies in which athletes assessed their coaches' servant leadership revealed that Rieke et al. (2008); Vidic and Burton (2011); Gillham et al. (2015), and Wang et al. (2021) all applied the RSLP-S. However, in the absence of studies that have followed procedures from item collection to scale development in the sport context, sport researchers have used various servant leadership scales developed for other contexts. For example, Peachey et al. (2018) and Lee (2019) used Ehrhart's (2004) single-factor scale. Lee et al. (2018) used Barbuto and Wheeler's (2006) scale, and Burton et al. (2017) used Van Dierendonck and Nuijten's (2011) scale. Since no robust measurement scales are currently in place in the sport domain, servant leadership research in the sport domain is considered to be in Phase 2, as Eva et al. (2019) have pointed out. In addition to servant leadership research targeting athletes, research on coaches (Dahlin and Schroeder, 2021; Robinson et al., 2021; Vinson and Parker, 2021) and employees of sport organizations (Megheirkouni, 2020; Svensson et al., 2021; Swanson et al., 2022) has also accumulated.

One of the major issues in servant leadership research is the lack of a definition (Eva et al., 2019). Since Greenleaf provided no clear definition, each researcher has used definitions and scales that align with their claims (Eva et al., 2019). Therefore, no consensus has been reached regarding the definition of servant leadership (Burton and Peachey, 2013). Eva et al. (2019) define servant leadership as “an (1) other-oriented approach to leadership (2) manifested through one-on-one prioritizing of follower individual needs and interests, (3) and outwards reorienting of their concern for self toward concern for others within the organization and the larger community (p.114).” This definition reflects the three characteristics that constitute servant leadership (i.e., motive, mode, and mindset). First, servant leaders are externally rather than internally motivated (Eva et al., 2019). Second, the servant leader mode reflects the perception that each follower is unique and has a different personality (Eva et al., 2019). Third, the servant leader mindset supports the growth of both followers and resources within the organization (Eva et al., 2019). Furthermore, servant leaders invest in the growth and wellbeing of others for the common good (Page and Wong, 2000) while promoting their followers' growth (Greenleaf, 2002), and providing services (Sendjaya, 2016). In the sport context (particularly, youth and college sport), “followers” are athletes, and the “common good” is considered to be the team's goal and purpose. Thus, in this study, coaching servant leadership is defined as an athlete-first approach to leadership that prioritizes athletes' needs and interests and serves them for a common goal of the team by investing in their growth and wellbeing.

While various scales have been developed by multiple researchers to measure servant leadership, Van Dierendonck (2011) identified six characteristics that constitute servant leadership based on a literature review: empowering and developing people, humility, authenticity, interpersonal acceptance, providing direction, and stewardship. Modifying the words to suit the sport context, each characteristic may be defined as follows (Van Dierendonck, 2011):

Empowering and developing people: motivation that focuses on enabling athletes, respecting them, and encouraging their growth.

Humility: the ability to put one's achievements and talents in an appropriate perspective and willingness to learn from others. It also demonstrates to athletes through words and actions that meeting their needs is a priority.

Authenticity: expressing oneself in line with one's thoughts and feelings and a commitment to honesty and self-responsibility.

Interpersonal acceptance: cognitive acceptance of athletes and demonstration of warmth and compassion.

Providing direction: possessing knowledge of the sport and coaching protocol and providing athletes with appropriate direction; creating new methods and approaches to old problems.

Stewardship: willingness to take responsibility and serve the team and athletes on behalf of one's self-interest. It also includes behavior that serves as a normative model for athletes.

Based on the above review, this study aimed to develop a coaching servant leadership scale in four phases following Hinkin's (1995) guidelines. In Phase 1, both deductive and inductive approaches were applied to collect items that potentially constitute coaching servant leadership. In Phase 2, the items' content validity was examined using Lawshe's (1975) content validity ratio. When Phases 1 and 2 were conducted, the items were categorized based on Van Dierendonck's (2011) classification and definitions. Phase 3 included exploratory factor analysis (EFA) and confirmatory factor analysis (CFA) to determine the factor structure of the measurement scale and to examine its fit with the data. Construct validity was further tested by assessing convergent validity, discriminant validity, and criterion-related validity. In Phase 4, the coaching servant leadership scale was replicated in another country (i.e., the United States) to enhance its generalizability.

## Methods

### Item Generation (Phase 1)

The inductive ten-statement testing method was applied with reference to Ito and Walker (2014) and Laub (1999). Ito and Walker (2014) asked “What is leisure?” and required participants to complete ten statements to collect leisure-related data. Laub (1999) asked participants “What do you judge to be the characteristics of a servant leader?” and had them identify ten characteristics. In the present study, the participants were asked, “What characteristics of a coach do you judge to indicate that they are a servant leader?” The participants consisted of 103 coaches and 34 university students who majored in sport pedagogy, sport psychology, or sport management at the author's university. The coaches were surveyed by the online survey panelists of a major survey company in Japan. Of the coaches' sample, 80.6% of the respondents were male (female = 19.4%) and their average age was 42.6 years (*SD* = 13.1).

For the deductive approach, items were collected from previous studies (Liden et al., 2008; Sendjaya et al., 2008; Van Dierendonck and Nuijten, 2011) recommended by Eva et al. (2019) and the previous study (Hammermeister et al., 2008) that have been shown to be applicable to sport. The statements obtained from both approaches were classified based on the six characteristics proposed by Van Dierendonck (2011). An expert review was conducted by the author and two doctoral students specializing in leadership and repeated until a consensus was reached.

### Content Adequacy Assessment (Phase 2)

To test the content validity of the items obtained in Phase 1, the content validity ratio (CVR) was calculated using responses from the experts following the method used by Lawshe (1975) and Grant and Davis (1997). The panel experts consisted of one associate professor, one assistant professor, and eight doctoral students. They were explained the definitions of servant leadership and the six characteristics by the author, and rated how well each item represented the characteristics using a four-point scale from 1 (it is not appropriate to measure this characteristic on this item) to 4 (it is appropriate to measure this characteristic on this item). The CVR value can range from −1 to +1, and a value of 0 means that half of the respondents rated the item as necessary. If the number of respondents is 10, a minimum CVR of 0.62 is required to satisfy the five percent level (Lawshe, 1975).

### Factor Analysis and Construct Validity Testing (Phase 3)

The sample for Phase 3 was assembled by online survey panelists from a survey company in Japan. A total of 936 high school athletes belonging to high school athletic clubs participated in the survey. In the screening prior to the survey, the participants were confirmed as members of their high school athletic clubs, with head coaches who coached them regularly. The mean age of the sample was 16.8 years (*SD* = 0.87; 36.4% male and 63.6% female). Sports comprising more than 5% of the sample included basketball (*n* = 124), soccer (*n* = 90), volleyball (*n* = 86), track and field (*n* = 79), badminton (*n* = 77), baseball (*n* = 53), and tennis (*n* = 48).

The sample was randomly split into two datasets to conduct EFA and CFA. As a general rule when conducting EFA, the sample size required is five to seven times the number of variables to be analyzed (Hair et al., 2010; Terwee et al., 2012). In this study, considering the number of samples obtained, the ratio was set at 7:1. Thus, the first dataset (*n* = 518) was used for EFA and the second (*n* = 418) was used for CFA. In the EFA, the appropriateness of factor analysis was examined based on two criteria: the Kaiser–Meyer—Olkin sampling statistic (KMO) and Bartlett's test of sphericity (BTS). According to Hair et al. (2010), the KMO value ranges from 0 to 1 and is close to 1 if each variable is predicted without errors associated with other variables: a value of 0.80 or above is considered meritorious. The BTS shows the statistical significance of significant correlations between several variables in the correlation matrix. EFA was conducted using maximum likelihood estimation with the promax rotation method.

For CFA, based on Kline's (2016) recommendation, the model fit was evaluated using the following fit indices: the normed chi-square (χ^2^/*df*), comparative fit index (CFI), root mean square error of approximation (RMSEA), standardized root mean square residual (SRMR), and Akaike information criterion (AIC). According to Hair et al. (2010), χ^2^/*df* ratios of 3:1 or less are associated with a better-fitting model; CFI values above 0.90 are associated with a model that fits well; RMSEA values of <0.08 are considered good; and SRMR values of 0.08 or less are associated with a good fit. Convergent validity was also confirmed based on the criteria that construct reliability (CR) is 0.70 or more and average variance extracted (AVE) is 0.50 or more. Discriminant validity was examined by comparing each factor's AVE and the square of the correlation between factors.

Of the 936 respondents, 687 proceeded to the next survey; that is, the data for the items on coaching servant leadership and the other variables used to test criterion-related validity were obtained from separate surveys. Hinkin (1998) suggested that criterion-related validity can be confirmed by examining the relationships between new measures and other variables that are theoretically correlated with them. In a review of existing research, Eva et al. (2019) identified behavioral outcomes (e.g., organizational citizenship behavior), attitudinal outcomes (e.g., commitment), leader-related outcomes (e.g., trust in the leader), and performance outcomes (e.g., team performance) as outcome variables of servant leadership. Team citizenship behavior (Martínez, 2013; Martínez and Tindale, 2015), team commitment (Kim et al., 2016), satisfaction with a head coach (Myers et al., 2011), and team efficacy (Bruton et al., 2016) were examined with respect to their relationships in the sport context. Team citizenship behavior was measured using 13 items (e.g., “I encourage other teammates when they are down;” α = 0.82); team commitment was measured using five items (e.g., “I would be very happy to spend the rest of my school years with this team;” α = 0.79), satisfaction with a head coach was measured using three items (e.g., “How much do you like playing for your coach?;” α = 0.83), and team efficacy was measured using one item (i.e., “Rate your team's confidence in their ability to perform to a high level sufficient to achieve success in their next competitive performance”). The participants scored all variables except team efficacy on a seven-point Likert scale; team efficacy was rated on a scale between 0 (not at all confident) and 100 (completely confident). The analyses were conducted using IBM SPSS Statistics 24 and IBM SPSS Amos 24.

### Replication in Another Country (Phase 4)

In Phase 4, an online survey was conducted in the United States among 278 athletes (113 males and 165 females) who belonged to an athletic club at a university. They ranged in age from 18 to 25 years (mean = 20.80 ± 1.83 years), and were freshmen (*n* = 42, 15.1%), sophomores (*n* = 62, 22.3%), juniors (*n* = 76, 27.3%), seniors (*n* = 93, 33.5%), or fifth-years (*n* = 5, 1.8%). Their teams belonged to the National Collegiate Athletic Association (NCAA) (*n* = 163), the United States Collegiate Athletic Association (USCAA) (*n* = 32), the National Junior College Athletic Association (NJCAA) (*n* = 27), the National Association of Intercollegiate Athletics (NAIA) (*n* = 18), and others. They also participated in Division 1 (*n* = 87), Division 2 (*n* = 135), and Division 3 (*n* = 56). Sports that comprised more than 5% of the sample included basketball (*n* = 70), soccer (*n* = 38), indoor volleyball (*n* = 29), football (*n* = 25), and tennis (*n* = 18).

Coaching servant leadership was measured using 17 items obtained from the results of Phase 3. Since the surveys in Phase 3 were conducted in Japanese, a back-translation procedure was performed before proceeding to Phase 4. First, the author translated the measurement items into English. Second, another researcher in leadership—a native speaker of Japanese who is fluent in English—translated them from English into Japanese. Finally, the original and translated items were examined separately by a professional translator who is a native speaker of Japanese and a native speaker of English, and the linguistic validity was confirmed.

## Results

### Item Generation (Phase 1)

**Figure 1:**
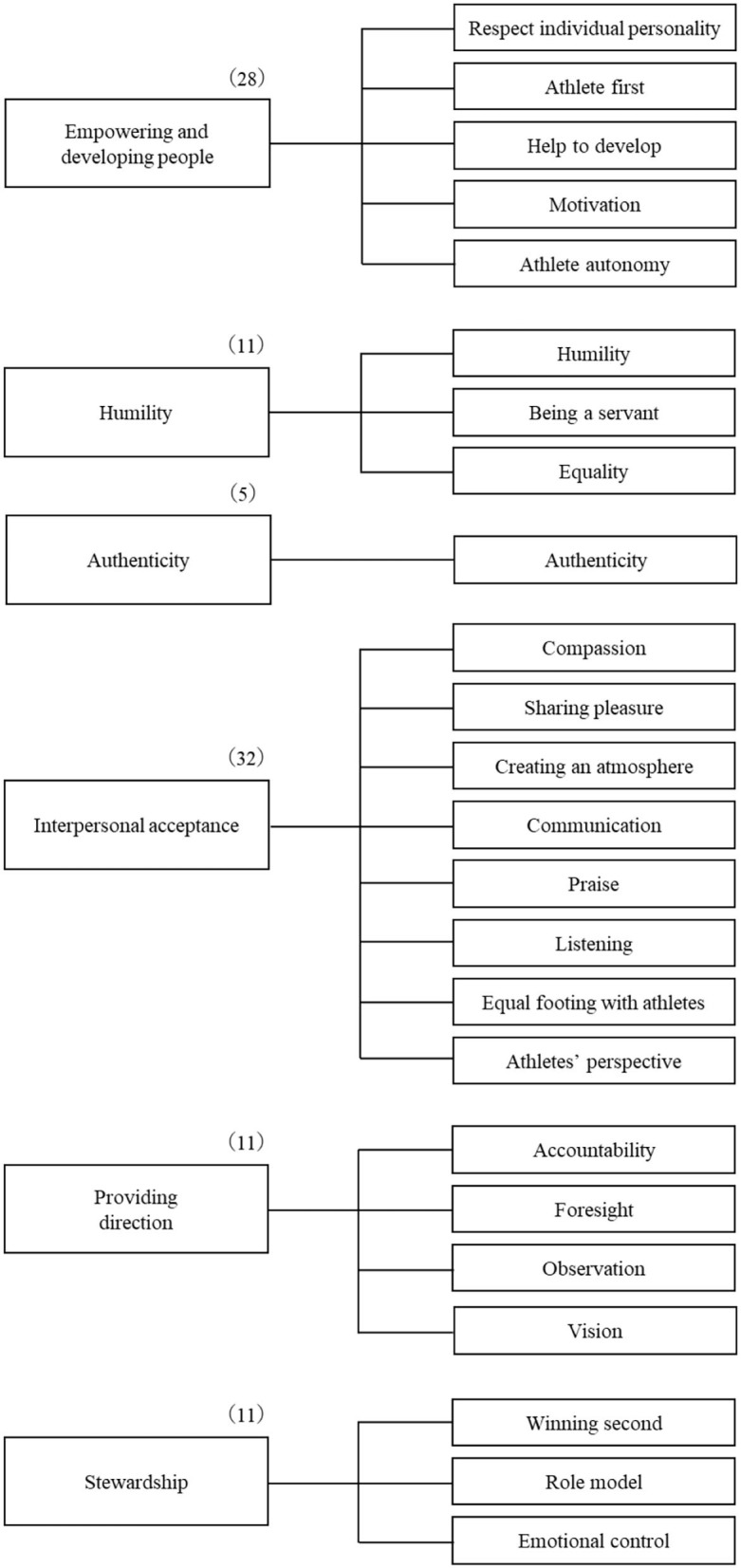
Classification of qualitative data based on the six categories proposed by Van Dierendonck (2011). Each number in parentheses indicates the number of items collected.

The items were collected using both a deductive (i.e., literature review) and inductive (i.e., surveys on coaches and university students) approach, and 844 statements were extracted, removing items with overlapping meanings unrelated to servant leadership. The 98 items obtained were grouped and classified into Van Dierendonck's (2011) six categories (see Figure 1).

### Content Adequacy Assessment (Phase 2)

To test content validity, the CVR was calculated based on the ratings of 10 panel experts. The criteria were not satisfied by 24 items, which were deleted. The wording of some items was modified, and two items were moved from “providing direction” to the “stewardship” category based on the panel experts' comments. At this point, 74 items were judged to be conceptually valid.

### Factor Analysis and Construct Validity Testing (Phase 3)

The EFA was conducted on dataset 1. First, the KMO (KMO = 0.96) and the BTS (*p* < 0.001) met the criteria, demonstrating the samples' appropriateness in dataset 1 for EFA. Next, based on Van Dierendonck's (2011) classification, the number of factors was fixed at six, and EFA was conducted using maximum likelihood estimation with the promax rotation method. Items with a minimum factor loading of < 0.5 and for which the difference in factor loadings between the two factors was < 0.2 were considered for deletion (Ferguson and Cox, 1993; Hair et al., 2010). Since the analysis was exploratory, strict criteria for item selection were deemed appropriate to create an efficient and reliable scale for use in this study and in future studies. The final factor analysis resulted in six factors with 17 items and explained 80.1% of the total variance. Each factor was named and defined as follows: acceptance (accepting athletes and creating an environment that facilitates communication; four items; α = 0.95), shared vision (sharing the team's goals with the athletes and providing them with clear direction; three items; α = 0.92), empowerment (drawing out the athletes' potential and encouraging their growth; three items; α = 0.92), dedication (prioritizing the athletes over oneself; three items; α = 0.92), humility (having the willingness to learn from others without overestimating oneself; two items; α = 0.80), winning second (being respectful to all involved in sport and contributing to the character building of athletes; two items; α = 0.90). The items, factor loadings, and descriptive statistics for coaching servant leadership are presented in Table 2. These 17 items across six factors were subject to CFA in dataset 2.

**Table 2:** Results of exploratory factor analysis and descriptive statistics.

	**1**	**2**	**3**	**4**	**5**	**6**	**Mean**	**SD**	**Skewness**	**Kurtosis**
**Acceptance (α = 0.95)**										
My head coach makes it easy for athletes to communicate with him/her (A1)	**0.98**	0.05	0.05	−0.11	−0.02	−0.03	4.24	2.06	−0.23	−1.24
My head coach can see things from the athletes' perspective (A2)	**0.76**	0.09	0.11	−0.06	−0.04	0.08	4.39	2.04	−0.35	−1.11
My head coach finds time to listen to the athletes' concerns (A3)	**0.68**	0.04	−0.01	0.21	0.02	0.03	4.57	1.97	−0.46	−0.96
My head coach proactively listens to the athletes' opinions (A3)	**0.67**	0.00	−0.03	0.18	0.08	0.07	4.60	2.01	−0.46	−1.00
**Shared vision (α = 0.92)**										
My head coach shares the athletes' goals (SV1)	0.15	**0.91**	−0.04	0.00	0.01	−0.11	4.86	1.99	−0.70	−0.70
My head coach understands the goals of the team (SV2)	0.04	**0.70**	−0.02	0.07	0.12	0.07	5.03	1.93	−0.88	−0.36
My head coach has a long-term vision for the team and not only short-term objectives (SV3)	−0.04	**0.68**	0.11	0.07	−0.07	0.17	4.95	1.93	−0.77	−0.53
**Empowerment (α = 0.92)**										
My head coach is aware of what limits the athletes' growth (E1)	0.12	0.02	**0.82**	0.03	0.01	−0.10	4.39	1.97	−0.37	−1.05
My head coach helps athletes realize their full potential (E2)	0.02	−0.02	**0.81**	−0.01	0.08	0.06	4.60	1.90	−0.50	−0.83
My head coach brings out the best in athletes (E3)	0.03	0.04	**0.68**	0.08	0.06	0.06	4.71	1.93	−0.59	−0.77
**Dedication (α = 0.92)**										
My head coach is happy to spend his/her private time helping the athletes practice (D1)	−0.12	0.25	0.01	**0.78**	−0.05	−0.01	4.76	1.93	−0.52	−0.85
My head coach puts the athletes' needs and interests ahead of his/her own (D2)	0.20	−0.08	−0.04	**0.77**	0.06	0.05	4.52	1.86	−0.39	−0.86
My head coach supports the athletes no matter what situation they are in (D3)	0.07	0.00	0.14	**0.75**	0.00	−0.01	4.66	1.87	−0.48	−0.80
**Humility (α = 0.80)**										
My head coach understands his/her weaknesses (H1)	−0.02	−0.02	0.01	−0.04	**0.80**	0.05	4.20	1.87	−0.20	−0.95
My head coach learns from criticism and failures (H2)	0.02	0.08	0.11	0.05	**0.68**	−0.05	4.48	1.90	−0.39	−0.91
**Winning second (α = 0.90)**										
My head coach values sportspersonship more than winning (WS1)	0.06	−0.01	−0.05	−0.02	0.05	**0.90**	4.77	1.96	−0.62	−0.77
My head coach provides athletes with opportunities to learn, even if there are no immediate results (WS2)	0.05	0.11	0.09	0.07	−0.04	**0.67**	4.94	1.90	−0.76	−0.51

*Factor loadings > 0.50 are in boldface*.

To test whether the factor structure obtained by the EFA fit the data, CFA was conducted using dataset 2 (see Table 3). First, a one-factor model with all items loaded on one factor was tested. The chi-square was 1190.15, *df* = 119, χ^2^/*df* = 10.00, CFI = 0.85, RMSEA = 0.15, SRMR = 0.05, AIC = 1,258.15. Next, the six-factor first-order model was tested and showed a satisfactory fit: χ^2^ = 246.85, *df* = 120, χ^2^/*df* = 2.06, CFI = 0.98, RMSEA = 0.05, SRMR = 0.02, AIC = 348.852. Comparison of the one-factor and six-factor first-order models revealed that the six-factor first-order model was reasonable for the coaching servant leadership scale [Δχ^2^(Δ*df*) = 943.30(1), *p* < 0.001].

**Table 3:** Summary of model comparisons.

**Models**	**χ^2^(*df*)**	**χ^2^/*df***	**Δχ^2^(Δ*df*)**	**CFI**	**RMSEA**	**SRMR**	**AIC**
Six-Factor first-order	246.85(120)	2.06	–	0.98	0.05	0.02	348.852
Six-Factor second-order	271.02(113)	2.34	24.17(7)	0.98	0.06	0.03	351.021
One-Factor	1,190.15(119)	10.00	943.30***(1)	0.85	0.15	0.05	1,258.15

*The six-factor second-order model and one-factor model are compared to the six-factor first-order model*.

*** *p < 0.001*.

The convergent and discriminant validity of the six-factor first-order model was examined. As presented in Table 4, all standardized factor loadings were above 0.70, ranging from 0.77 to 0.92. The CR ranged from 0.84 to 0.94, and the AVE ranged from 0.72 to 0.81 within each factor. Thus, convergent validity was established. Furthermore, comparing the AVE scores with the squared correlations between the two factors indicated that all factors exhibited discriminant validity (see Table 5). However, since highly correlated latent factors were found, the six-factor first-order and six-factor second-order models were compared. As Table 3 illustrates, the six-factor second-order model exhibited an adequate fit: χ^2^ = 271.02, *df* = 113, χ^2^/*df* = 2.34, CFI = 0.98, RMSEA = 0.06, SRMR = 0.03, AIC = 351.021. The first-order factors loaded significantly on the second-order factor (*p* < 0.001) and ranged from.87 to.92. Although no significant difference emerged in the Δχ^2^ test, the six-factor first-order model showed a slightly better fit to the data than the six-factor second-order model. Even if the second-order factor model could effectively explain the model, it would not show a better fit than the first-order factor model (Marsh and Hocevar, 1985). Earlier studies tended to explain servant leadership using a second-order factor model (Sendjaya and Cooper, 2011; Zhang et al., 2016), and since the factor correlations in this study were also high, a second-order factor model was considered better for explaining coaching servant leadership.

**Table 4:** Results of confirmatory factor analysis for the coaching servant leadership scale.

**Factors**	**Items**	**FL**	**CR**	**AVE**
Acceptance	A1	0.90	0.94	0.80
	A2	0.88		
	A3	0.89		
	A4	0.91		
Shared vision	SV1	0.91	0.93	0.81
	SV2	0.92		
	SV3	0.87		
Empowerment	E1	0.86	0.92	0.79
	E2	0.90		
	E3	0.90		
Dedication	D1	0.84	0.91	0.77
	D2	0.90		
	D3	0.90		
Humility	H1	0.77	0.84	0.72
	H2	0.92		
Winning second	WS1	0.85	0.85	0.74
	WS2	0.88		

*FL, factor loadings; CR, construct reliability; AVE, average variance extracted*.

**Table 5:** Average variance extracted, correlations, and squared correlations.

	**A**	**SV**	**E**	**D**	**H**	**WS**
A	**0.80**	0.63	0.72	0.67	0.66	0.71
SV	0.79	**0.81**	0.66	0.60	0.54	0.70
E	0.85	0.81	**0.79**	0.68	0.64	0.66
D	0.82	0.77	0.82	**0.77**	0.68	0.56
H	0.81	0.73	0.80	0.82	**0.72**	0.51
WS	0.84	0.84	0.82	0.75	0.71	**0.74**

*A, acceptance; SV, shared vision; E, empowerment; D, dedication; H, humility; WS, winning second*.

*Bold values in diagonal are the average variance extracted; values below the diagonal are correlations; values above the diagonal are squared correlations*.

*All correlations significant p < 0.001*.

To test criterion-related validity, the correlations between each coaching servant leadership variable and the four outcome variables were calculated (see Table 6). The results indicated that all coaching servant leadership variables were correlated with four outcomes. Satisfaction with a head coach showed the strongest relationship, with correlations ranging from 0.60 to 0.69. Thus, the criterion-related validity of the coaching servant leadership scale was confirmed.

**Table 6:** Correlations between coaching servant leadership and outcome variables.

	**S**	**TCB**	**TC**	**TE**
Acceptance	0.69***	0.46***	0.32***	0.31***
Shared vision	0.67***	0.43***	0.34***	0.37***
Empowerment	0.69***	0.43***	0.36***	0.35***
Dedication	0.62***	0.44***	0.29***	0.34***
Humility	0.60***	0.33***	0.27***	0.27***
Winning second	0.62***	0.46***	0.30***	0.27***

*S, satisfaction with a head coach; TCB, team citizenship behavior; TC, team commitment; TE, team efficacy*.

*** *p < 0.001*.

### Replication in Another Country (Phase 4)

The scale's generalizability was tested via CFA among university athletes in the United States. Since the second-order factor model was confirmed appropriate in Phase 3, CFA was performed with coaching servant leadership as the second-order factor and indicated that the overall fit of the six-factor second-order model was satisfactory: χ^2^ = 192.85, *df* = 113, χ^2^/*df* = 1.71, CFI = 0.97, RMSEA = 0.05, SRMR = 0.04. The first-order factors loaded significantly on the second-order factor (*p* < 0.001) and ranged from 0.76 to 0.94. The standardized factor loadings from the first-order factors to the observed variables ranged from 0.63 to 0.87. The CR for each first-order factor ranged from 0.74 to 0.84. Next, the Δχ^2^ test was performed to compare the six-factor second-order model with the one-factor model. The test revealed that the six-factor second-order model was superior to the one-factor model [Δχ^2^(Δ*df*) = 164.95(6), *p* < 0.001].

As with Phase 3, the correlation coefficients between the six coaching servant leadership variables and the four outcome variables were calculated to test criterion-related validity. Satisfaction with a head coach (*r* = 0.37−0.60), team citizenship behavior (*r* = 0.49−0.59), and team commitment (*r* = 0.31−0.55) were significantly related to each coaching servant leadership variable. While team efficacy was not significantly related to humility (*r* = 0.11), it was significantly related to all other coaching servant leadership variables (*r* = 0.14−0.19). Hence, the criterion-related validity of coaching servant leadership was demonstrated in a sample of university athletes in the United States.

## Discussion

This study aimed to develop and test a coaching servant leadership scale. First, a pool of items was generated using deductive (i.e., literature review) and inductive (i.e., surveys among coaches and university students) approaches. Next, the items were narrowed down for the initial analysis based on experts' assessment of content validity. Third, after several items were removed by EFA, CFA supported the six-factor structure, providing evidence of construct validity. Fourth, the scale's criterion-related validity was established as the relationships between each coaching servant leadership variable and the four outcome variables were confirmed. Finally, a survey was conducted among university athletes in the United States to enhance the scale's generalizability. Consequently, a coaching servant leadership scale consisting of six factors was developed.

Based on Van Dierendonck's (2011) classification, the study's findings supported the reliability and validity of the coaching servant leadership scale. This scale also corresponds to Page and Wong's (2000) conceptual framework for measuring servant leadership [i.e., character-orientation (“What kind of person is the leader?”), people-orientation (“How does the leader relate to others?”), task-orientation (“What does the leader do?”), and process-orientation (“How does the leader impact organizational processes?”)]. Specifically, dedication and humility are included in character-orientation. Character-orientation concerns the development of a servant's attitude, focusing on the leader's values, credibility, and motivation (Page and Wong, 2000). A servant leader puts their accomplishments and talent into appropriate perspective and is willing to learn from others' expertise (Van Dierendonck, 2011). A servant leader also prioritizes the needs of others beyond their own and serves others to help them grow (Ehrhart, 2004; Liden et al., 2008). Those who are served are more likely to become servant leaders themselves (Greenleaf, 2007). Thus, humility and dedication are key factors in coaching servant leadership, both in terms of building the coach's own character and influencing athletes. Acceptance and empowerment correspond to people-orientation, which is related to the development of human resources. This concept focuses on how a leader relates to others and their commitment to developing them (Page and Wong, 2000). Van Dierendonck and Nuijten (2011) stated that acceptance is about empathy, which leads to high levels of relationship building. The core of servant leadership is its transformational influence on followers, which includes empowering them (Sendjaya et al., 2008). That is, a coach who implements servant leadership will warmly accept their athletes and create an environment conductive to communication. They can then motivate athletes to encourage their growth and autonomy. Shared vision overlaps with task-orientation, while winning second is related to process-orientation. Task-orientation focuses on leadership tasks and skills that are necessary for success. According to Page and Wong (2000), a servant leader engages the entire team in the process of creating a shared vision, encouraging each individual to apply their unique talents to achieve the vision autonomously. A shared vision also promotes teamwork (Page and Wong, 2000)—an essential element for sport teams. It can also be related to satisfying athletes' psychological needs and promoting athletes' intrinsic motivation, in accordance with self-determination theory (Deci and Ryan, 2002). Process-orientation focuses on the leader's ability to model and develop flexible, efficient, and open systems in the process of building an organization (Page and Wong, 2000). Winning second, one of the factors that constitute coaching servant leadership, appears to be unique to the sport field. This implies the need for an athlete-centered coaching philosophy and does not imply that striving to win is a bad thing. Coaches who are athlete-centered prioritize the athlete's physical and social development as well as their enjoyment (McGladrey et al., 2010). According to Walton ((n.d.)), John Wooden, the famous American basketball coach, derived his joy and happiness from the success of others. He said that he learned from Abraham Lincoln and Mother Teresa that “a life not lived for others is not a life.” This statement accurately reflects the ideals of servant leadership.

The present study confirmed that the correlations between the coaching servant leadership factors were high, while the one-factor model showed a poor fit with the data. Therefore, the second-order model's fit with the data was as good as that of the first-order model. This result is consistent with earlier studies (Liden et al., 2008; Sendjaya and Cooper, 2011; Van Dierendonck and Nuijten, 2011): the evidence demonstrates that coaching servant leadership is a hierarchical model that is captured by holistic and multi-dimensional constructs.

Criterion-related validity testing of the scale revealed significant positive correlations between each coaching servant leadership factor and the four outcome variables identified by Eva et al. (2019), with the exception of humility and team efficacy in the US sample. This result indicates that coaching servant leadership can be positively related to various psychological aspects of athletes. Existing research also reports that servant leader coaches have a more positive impact on athletes' satisfaction, intrinsic motivation, and mental skills than non-servant leader coaches (Rieke et al., 2008). Since servant leadership research in the sport domain lags behind business management, further research on its impact on athletes is recommended as an avenue for future study.

### Practical Implications

This study's findings indicate several important practical implications that are beneficial to the coaching research and coaching scene. First, this study identified the factors of coaching servant leadership that positively impact athletes' growth and wellbeing. In addition to the servant leadership of coaches identified by Hammermeister et al. (2008), the identification of new factors in this study represents a major step forward in sport leadership research. As mentioned earlier, winning is not everything for athletes and sport teams. Although striving to win is not wholly bad, excessive emphasis on victory increases the likelihood that multiple ethical issues will arise. Servant leaders who focus on athletes reportedly contribute to the creation of an ethical climate in sport organizations (Burton et al., 2017). Thus, coaches are encouraged to refer to this scale as a means of learning about coaching servant leadership with the aim of applying it to developing athletes and building teams.

Second, the study revealed the relationships between coaching servant leadership and various athlete variables. This finding indicates that coaching servant leadership is likely to have a positive impact on athletes in actual sport settings. Servant leadership is viewed with concern partly based on the fear that it will produce mentally weak athletes, which has been empirically refuted by Rieke et al. (2008). It is anticipated that coaching servant leadership will improve coaching quality and provide coaches with opportunities to build good relationships with athletes.

### Limitations and Future Research

Several limitations must be considered in interpreting the present study's results. First, an online survey was conducted to assemble a large sample of athletes. Although various data were obtained, these data may have been biased. Furthermore, since no multi-level data could be collected, team-level analysis was not possible. Future studies should collect samples at the team-level and apply multi-level analysis to examinations of coaching servant leadership. Moreover, it would be desirable to study not only youth and university sport but also athletes and coaches in other contexts, such as sport clubs. Second, although the study was preceded by as many careful procedures as possible, the present study alone does not necessarily verify that the coaching servant leadership scale, consisting of six factors and 17 items, is a robust instrument. Earlier servant leadership studies have reconsidered the number of factors and developed short forms (Liden et al., 2015; Sendjaya et al., 2019). Further research may be required to examine and refine this matter.

In conclusion, the present study is the first in the sport domain to apply both deductive and inductive approaches to generate items and develop a coaching servant leadership scale. The scale's applicability was confirmed not only for Japan but also for the United States. This finding contributes considerably to servant leadership research in the sport domain. The accumulation of future research, including empirical examination of the theoretical models and their application to coaching practice, is anticipated.

## Data Availability Statement

The raw data supporting the conclusions of this article will be made available by the authors, without undue reservation.

## Ethics Statement

The studies involving human participants were reviewed and approved by the Research Ethics Board of Kobe Shinwa Women's University. Written informed consent from the participants' legal guardian/next of kin was not required to participate in this study in accordance with the national legislation and the institutional requirements.

## Author Contributions

The author confirms being the sole contributor of this work and has approved it for publication.

## Funding

This work was supported by JSPS KAKENHI Grant No. 19K19966.

## Conflict of Interest

The author declares that the research was conducted in the absence of any commercial or financial relationships that could be construed as a potential conflict of interest.

## Publisher's Note

All claims expressed in this article are solely those of the authors and do not necessarily represent those of their affiliated organizations, or those of the publisher, the editors and the reviewers. Any product that may be evaluated in this article, or claim that may be made by its manufacturer, is not guaranteed or endorsed by the publisher.
